# Pulmonary adenocarcinoma metastasis to a dorsal root ganglion: a case report and review of the literature

**DOI:** 10.1186/1752-1947-7-212

**Published:** 2013-08-23

**Authors:** Philipp Jörg Slotty, Jan Frederick Cornelius, Timo Marcel Schneiderhan, Kamp Marcel Alexander, Richard Bostelmann

**Affiliations:** 1 Neurochirurgische Klinik, Heinrich-Heine-Universität Düsseldorf, Moorenstrasse 5, Düsseldorf 40225, Germany; 2 Institut für Neuropathologie, Heinrich-Heine-Universität Düsseldorf, Moorenstrasse 5, Düsseldorf 40225, Germany

## Abstract

**Introduction:**

The dorsal root ganglion is a rare manifestation of metastatic spread. We report what we believe to be the first case of metastasis of a pulmonary adenocarcinoma to the lumbar dorsal root ganglion. Only four descriptions for different primary tumors spreading to the dorsal root ganglion have been described in the literature so far.

**Case presentation:**

A 70-year-old Caucasian woman with a four-month history of left-sided lumbar radiculopathy was admitted to our department under the assumption of a herniated lumbar disc. Her past medical history included a pulmonary adenocarcinoma and invasive ductal breast cancer.

Lumbar magnetic resonance imaging revealed a space-occupying mass in her left neuroforamen L3-L4 with compression of her L3 nerve root. Neurinoma was taken into account as a differential diagnosis, although not considered typical. Surgery revealed a metastasis of pulmonary adenocarcinoma to her dorsal root ganglion.

**Conclusions:**

Dorsal root ganglion metastases seem to be extremely rare and can mimic primary local nerve sheath tumors. Therefore, they usually present as incidental findings. Resection should be performed strictly under intraoperative monitoring as tumor spread between the nerve fibers is commonly observed. Metastases should be taken into account in spinal nerve tumors involving the dorsal root ganglion, especially in patients harboring known malignant diseases. The low incidence means that no clear treatment advice can be given. Resection is possible under intraoperative monitoring and relieves neurological symptoms.

## Introduction

Dorsal root ganglion (DRG) metastasis has been rarely described in the literature. Up to now, only four cases in three publications are listed, comprising two cases from autopsy series and two case reports. The underlying pathologies include ductal breast cancer, pulmonary oat-cell cancer, renal cell carcinoma and uterine carcinoma [1-3]. Although metastatic spread to the central nervous system is common in pulmonary adenocarcinoma, DRG metastasis has not yet been described. We report what we believe to be the first case of an incidental DRG metastasis of a pulmonary adenocarcinoma.

## Case presentation

A 70-year-old Caucasian woman with a four-month history of left-sided lumbar radiculopathy was admitted to our department under the assumption of a herniated lumbar disc. Her current pain level was 7 out of 10 on the visual analogue scale. Walking was still possible using crutches. In a clinical examination, a left-sided hip flexor paresis, paresthesia and numbness in the distribution of her left L3, and a loss of the patellar reflex were seen. Her past medical history included a pulmonary adenocarcinoma (T2 N1 G2 R0 M0) treated by partial pulmonary resection on her left side 13 years before, and invasive ductal breast cancer (T1b N0 G2 R0, human epidermal growth factor receptor 2-negative), treated by right-side mastectomy and sentinel lymph node dissection five years before. In clinical follow-up, no local recurrence or metastases were seen.

**Figure 1:**
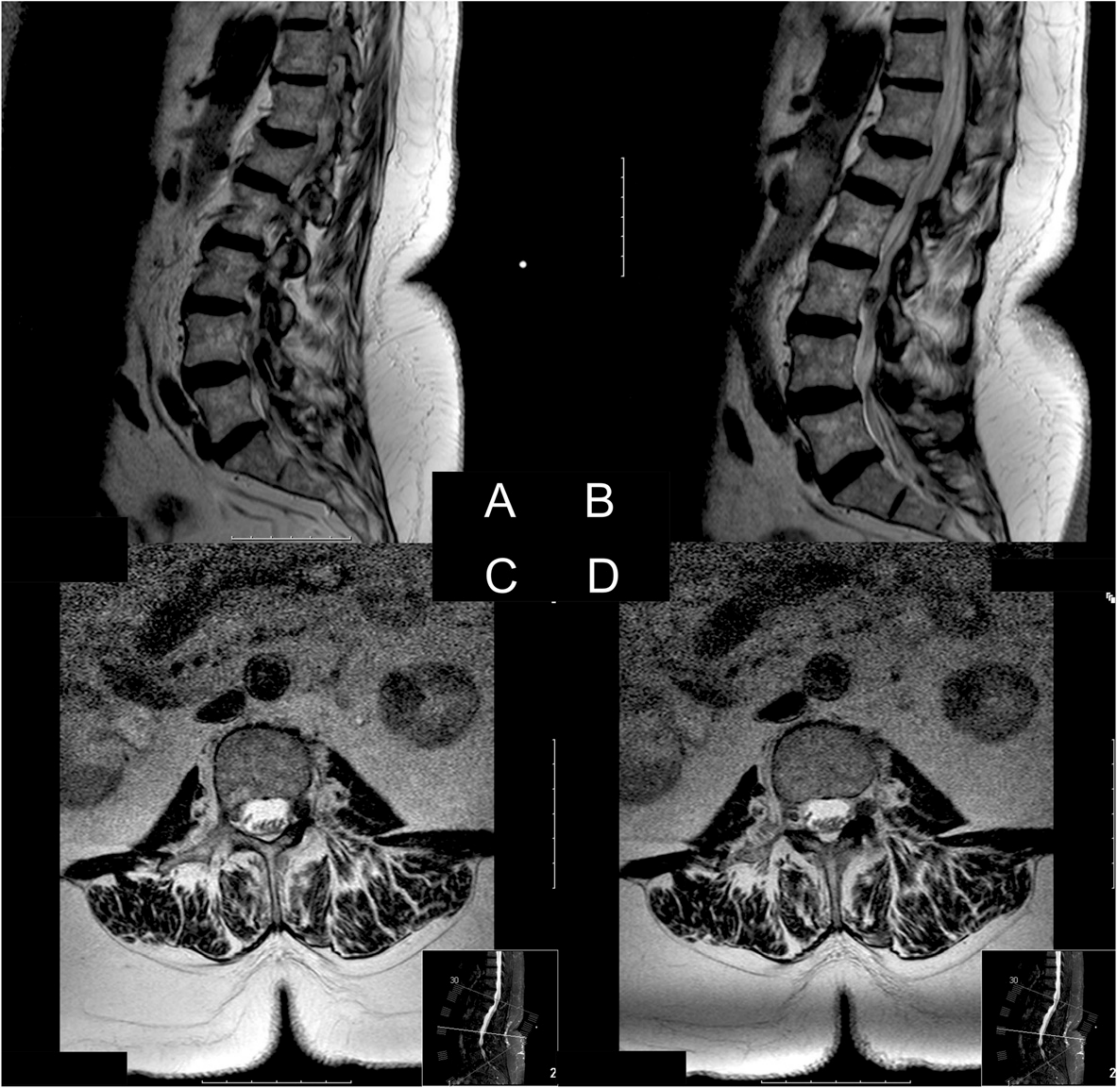
**Preoperative magnetic resonance imaging. (A)** Sagittal non-enhanced T1-imaging. **(B)** Sagittal T2-imaging. **(C, D)** Axial T2-imaging. An unclear structure in the neuroforamen of the L3 left nerve root not clearly delimitable. There was no clear connection to the intervertebral disc space. No gadolinium was applied because of renal insufficiency.

Lumbar magnetic resonance imaging revealed a space-occupying mass in her left neuroforamen L3-L4 with compression of the L3 nerve root (Figure 1). Gadolinium application was not possible because of renal insufficiency. Radiological differential diagnosis included a herniated lumbar disc or neurinoma. Conservative treatment of pain control failed and the accompanying progressive neurologic deficits required that our patient be admitted for herniated lumbar disc surgery. Neurinoma was taken into account as a differential diagnosis, although we did not consider the radiographical findings typical.

**Figure 2:**
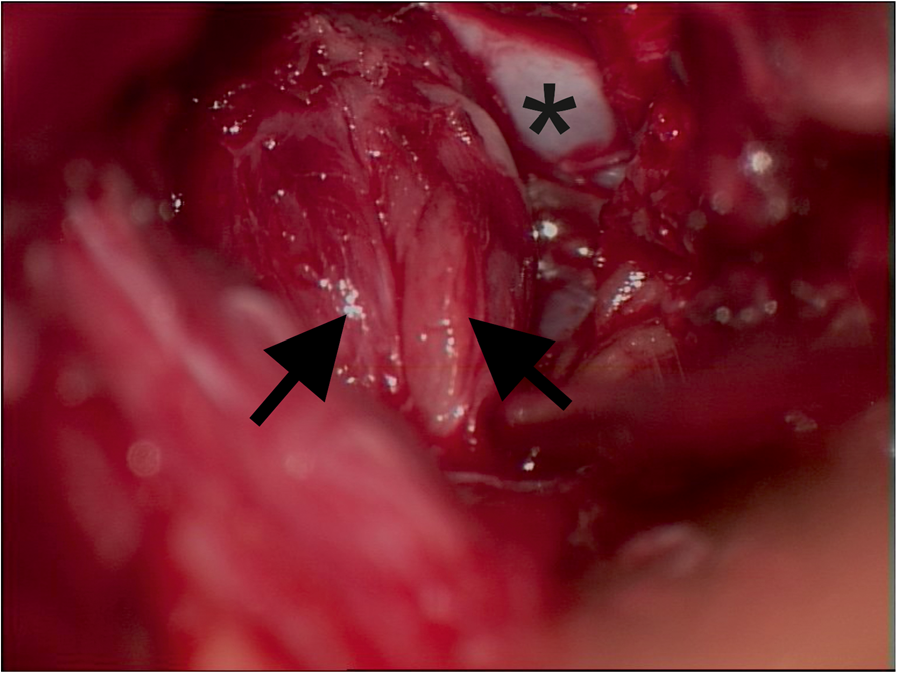
**Intraoperative view from lateral into the left neuroforamen L3-L4.** Shows the aspect of the swollen L3 ganglion. The dural sheath was thinned out by the process and nerve fibers (large arrows) are splayed by the process with no clear delineation.*Ventral osseous wall of the neuroforamen.

Due to the intraforaminal location of the lesion, a dorsolateral minimal invasive approach was used. Following conservative decompression and opening of the neuroforamen, only residual dural sheath was found. An unclear structure filling her neuroforamen was identified as a swollen ganglion by following its course. Nerve fibers were splayed with interjacent dense tissue (Figure 2). No salient vascularization was found. Cryosection was performed and examination of the resulting specimen showed a malignant non-nerve sheath tumor of unknown origin. Intraoperative monitoring of somatosensory evoked potentials and motor evoked potentials with direct *in situ* stimulation signaled disseminated active fibers in the tissue, impeding further dissection and complete tumor removal. An extended decompression of the ganglion and nerve root was performed following subtotal tumor removal.

Our patient recovered in time and had no complaints of radicular pain post-surgery. Her flexion paresis was not completely normalized but significantly improved.

**Figure 3:**
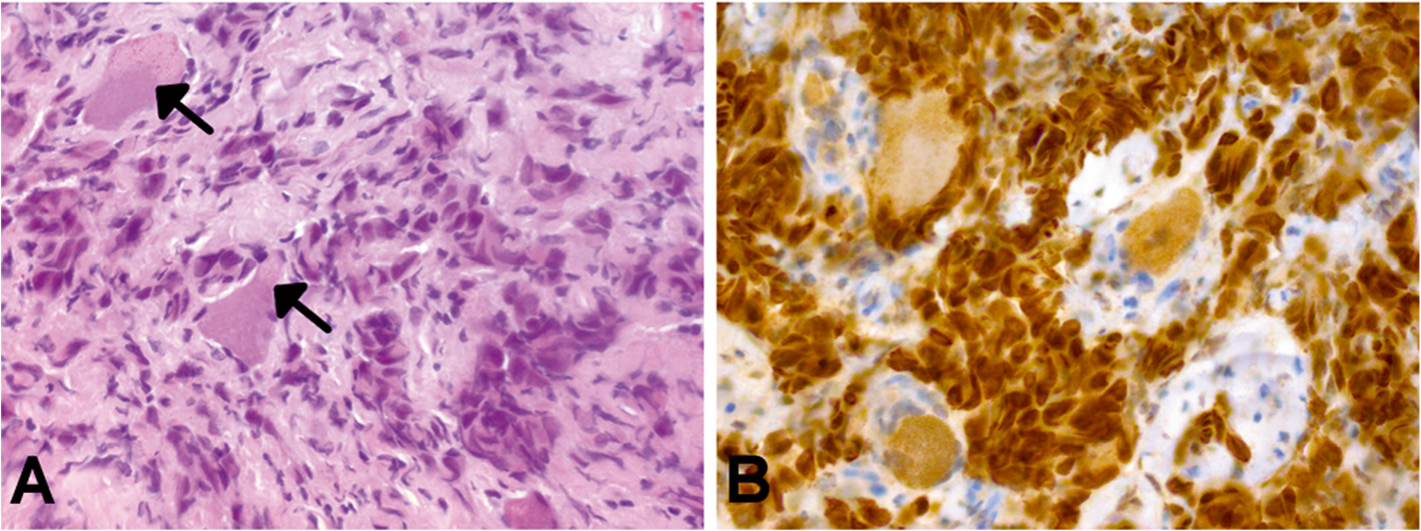
**Histological findings. (A)** Small nests of epithelial tumor cells growing within neuronal tissue (hematoxylin-eosin staining). Note the large ganglion cells (arrows). **(B)** On immunohistochemistry, the tumor cells stain strongly positive for thyroid transcription factor 1, suggesting origin from a lung carcinoma. Original magnification of each picture: ×400.

A histological examination revealed a metastatic lesion consisting of epithelial tumor cells, which grow in small nests between large ganglion cells (Figure 3). Immunohistochemical staining showed positivity for cytokeratins 7 and 8 as well as thyroid transcription factor 1, suggesting origin from a lung adenocarcinoma.

Staging included cerebral and whole spine magnetic resonance imaging. An additional C7 osseous metastasis and a muscular metastasis in her right gluteal were seen. No additional metastases in her central or peripheral nervous system were found. Following interdisciplinary discussion, our patient was referred for radiochemotherapy, including fractionated radiation of the L2-L5 segment with a cumulative dose of 40Gy. Despite intensive treatment, our patient developed rapid clinical deterioration in the following weeks and was diagnosed with meningeal carcinomatosis 12 weeks after surgery. She died two weeks later.

## Discussion

DRG metastases are rare and may be incidental findings in surgery for herniated discs, dorsal root neurinoma or spinal meningioma. This has to be taken into account in unanticipated intraoperative findings, including normal disc anatomy and a thinned dural sheath of the nerve root. A common finding in this pathology is enlargement of the ganglion with splaying of the nerve fibers. Metastasis of pulmonary adenocarcinoma to the DRG has not previously been described but cases of trigeminal ganglion metastasis have [4]. Our case report provides further information on the potential of this entity to spread to the border between the central and peripheral nerve systems.

No metastases to the nerve root itself have been described so far. The possible pathophysiological mechanisms of DRG metastasis were discussed in a description of a breast cancer DRG metastasis. Based on histopathological examinations, it was assumed that the fenestrated endothelium, in contrast to the non-fenestrated endothelium in the nerve root, facilitates DRG but not nerve root metastasis. However, the number of known cases is too small to detect similarities shared by the different entities described. It is noticeable that none of the patients described so far harbored or developed metastases in the central nervous system. DRG metastasis might require distinct genetic changes in the primary tumor to evolve.

Metastatic spread of different primary tumors into the DRG, including different species of carcinomas, has been described; however, it is likely that a significant number of cases remain undetected. Besides the low incidence of this entity, this may be explained by the reduction in neurologic deficits and pain due to nerve root decompression. In unclear cases, an exposition of the DRG with inspection and biopsy has to be performed. Although bone metastases of the spine with compression of neuronal structures are more common than DRG metastases, the latter should be considered as a rare differential diagnosis of a lumbar disc herniation or schwannoma for patients with a known malignancy and a radiological mass at the neuroforamen.

Surgery for these rare instances is complicated by their usually unanticipated occurrence. Lesions unclear on radiology, especially in patients with known malignancies, should result in an increase in presurgical diagnostic work-up, ideally including positron-emission tomography imaging. Intraoperative neurophysiological monitoring should be used in all unclear lesions. Previous reports indicate that clear delineation of tumor and nerve tissue or tumor capsules is usually not found, and subtotal resections were generally performed [3]. Complete tumor removal with preservation of neurological function is usually not an option. The benefit and importance of intraoperative direct nerve stimulation has been shown in benign and malignant peripheral nerve tumors [5]. Therefore, subtotal resection and nerve root decompression should be performed under neurophysiological monitoring to allow confirmation of the diagnosis and to improve clinical symptoms and quality of life in these patients. This is especially true with DRG metastases as they likely indicate systemic tumor spread and life expectancy is limited in these patients.

Advanced treatment options include resection of the process including the DRG with acceptance of neurologic deficts followed by local radiation therapy. We believe these options should be subject to the patients’ discretion. Radical surgery may then be considered following complete tumor staging, including cerebrospinal fluid cytology.

## Conclusions

DRG metastases are extremely rare. Different tumor entities seem to possess the potential for DRG metastasis. When there are inconclusive imaging and unusual intraoperative findings, rare causes have to be kept in mind, and not only in those patients with pre-existing malignancies. No clear treatment recommendation exists. Based on the few cases reported, nerve root decompression, subtotal resection and adjuvant treatments including local radiation seem to represent the best management option.

## Consent

Written informed consent was obtained from the patient’s relatives for publication of this case report and accompanying images. A copy of the written consent is available for review by the Editor-in-Chief of this journal.

## Abbreviations

DRG: Dorsal root ganglion.

## Competing interests

The authors declare that they have no competing interests.

## Authors’ contributions

PJS, JFC and KMA analyzed and interpreted the patient data including follow-up and reviewed the literature. PJS and RB were major contributors in writing the manuscript. TMS performed the histological examinations. All authors read and approved the final manuscript.
